# Age at time of surgery does not influence outcome in idiopathic normal pressure hydrocephalus – a national quality registry study of 3082 patients

**DOI:** 10.1007/s00701-025-06717-y

**Published:** 2025-12-01

**Authors:** C. Chidiac, N. Sundström, M. Tullberg, L. Arvidsson, M. Olivecrona

**Affiliations:** 1 Department of Neurosurgery, Sahlgrenska University Hospital, Gothenburg, Sweden; 2 Faculty of Medicine and Health, Örebro University, Örebro, Sweden; 3 Department of Diagnostics and Intervention, Radiation Physics, Biomedical Engineering, Umeå University, Umeå, Sweden; 4 Hydrocephalus Research Unit, Department of Clinical Neuroscience, Institute of Neuroscience and Physiology, Sahlgrenska Academy, University of Gothenburg, and Department of Neurology, Sahlgrenska University Hospital, Gothenburg, Sweden; 5 Department of Women’s and Children’s Health, Karolinska Institutet, Department of Neurosurgery, Karolinska University Hospital, Stockholm, Sweden; 6 Department of Neurosurgery, Faculty of Medicine and Health, Örebro University, Örebro, Sweden

**Keywords:** Idiopathic normal pressure hydrocephalus, Age, Shunt surgery, MRS, TUG

## Abstract

**Purpose:**

This study aims to investigate the outcome of shunt surgery in patients with idiopathic normal pressure hydrocephalus (iNPH) in relation to age at surgery, using data from the Swedish Hydrocephalus Quality Registry (SHQR). The disease affects older patients, with a mean age at diagnosis of 74 years. Since shunt surgery, which introduces a risk of serious complications, is the only available treatment, appropriate selection of patients eligible for surgery is essential. It has been suggested that higher age negatively affects outcome after shunt surgery.

**Methods:**

Patients operated upon during January 2004–February 2022 were included. The inclusion criteria were: age ≥ 60 years and data available from ≥ 3 domains by the iNPH scale. Clinical outcomes were assessed at 3 and 12 months using the modified version of the iNPH scale (miNPH), the Timed Up-and-Go (TUG) test and the modified Rankin scale (mRS). These were related to 5-year-interval age groups.

**Results:**

Improvement was seen in all age groups, with no statistically significant differences in outcome between age groups in miNPH score, TUG or mRS. The oldest group (> 85 years) showed significant improvements, as illustrated by miNPH scale score changes at 3 and 12 months of 4.3 (–8.1 to 21.5) and 10.1 (–6.5 to 36.8), respectively.

**Conclusions:**

This population-based study shows similarly favourable outcomes across ages, suggesting that there should be no upper age limit for shunt surgery in patients with iNPH.

## Introduction

Idiopathic normal pressure hydrocephalus (iNPH) is a progressive disease that mostly affects older adults. The treatment of choice is shunt surgery [27], and the mean age at surgery for iNPH in Sweden is 74 years, regardless of sex. One challenge in the treatment for iNPH is to offer surgery at an early stage, when it is as effective as possible, weighing benefits versus risks. It has been shown that prompt surgery after diagnosis is more advantageous than delayed surgery [1, 4]; moreover, non-deterioration must be seen as a benefit, regardless of improvement.

Increasing age has been reported as a negative prognostic factor for many neurosurgically treated diseases, such as glioblastomas, meningiomas and so forth [11, 16, 18, 29]. Some reports have shown that increasing age negatively influences outcome after shunt surgery for iNPH [12], while others find no negative influence of age on outcome in iNPH [2, 3].

Another aspect with a negative influence on outcome is frailty. It can be defined as an increasing time in the Timed Up-and-Go (TUG) test [9, 17, 21]. Several researchers have reported increasing frailty as a negative factor affecting outcome in several surgical conditions Some studies have reported that frailty negatively influences the outcome of shunt surgery for iNPH, while others have not found the same relation [12, 22].

The American Society for Anesthesiology (ASA)’s Physical Status Classification System [23] can be used as a surrogate measure of risk assessment for surgery [6, 20]. The incidence of chronic subdural haematoma, glioma and meningioma is increasing with the ageing population [5, 13, 14]. Löfgren et al. studied the outcome of glioma and meningioma surgery in an older population and found that neither the complication rate nor mortality was correlated with age [13, 14].

The mean age of death in Sweden was 84.9 years for women and 81.6 years for men in 2023 [15]. The remaining lifetime at a given (even advanced) age is expected to rise. In Sweden, the expected remaining mean survival time of women and men who had reached 80 years of age was 10.24 years and 8.78 years in 2024, respectively. By 2050, this expected remaining mean survival time is predicted to increase to 11.65 and 10.35 years [46]. With the world’s population above the age of 65 years growing more rapidly than the population below that age, the incidence of iNPH is expected to increase, along with the need for surgery to treat it [8]. Thus, it is essential to establish how age may influence outcome after surgery in iNPH.

The Swedish Hydrocephalus Quality Register (SHQR) is a national register including all patients operated upon for hydrocephalus. Dedicated personnel at each university hospital in Sweden are responsible for data transfer into the register. Data on standardised, clinical measurement scales used when diagnosing patients and when determining 3- and 12-month outcome are registered in the SHQR [24].

To the best of our knowledge, no previous national or international study has reported that more advanced age in iNPH patients at the time of shunt surgery affects the outcome in any direction.

## Aim

The aim of this study was to investigate the clinical outcome of shunt surgery in Sweden for patients with iNPH in relation to age at surgery.

## Materials and methods

### Patients

All patients registered in the SHQR during the period January 2004–February 2022 that were 60 years of age or older were eligible for inclusion in the study. Patients for whom less than three of the four symptom domains could be assessed pre- and post-operatively were excluded. Age is defined as age at the time of shunt surgery. TUG test results were first included in the SHQR in 2010. All neurosurgical departments follow up at 3 months, but not all have a 12-month follow-up due to work-up decided on at the departments in accordance with recourses etc. and/or lack of follow-up patients due to different reasons not stated in the registry.

### iNPH scale assessment of clinical symptoms and outcome

Baseline clinical status and outcome were defined using the iNPH scale, as introduced by Hellström et al., with pre- and post-operative assessments [7]. The scale consists of four variables, each of which corresponds to a symptom domain, summed up in an equation: (2 × gait + balance + incontinence + neuropsychology)/5 (or number of available domain scores). Each scale score for the four variables in the SHQR was converted into a continuous domain score ranging from 0 (most severe state) to 100 (performance of age-matched healthy population) [7]. The equation can be modified by reducing the denominator by the number of missing domains, resulting in a modified iNPH score (miNPH score). A significant improvement in the miNPH scale is defined as an increase of ≥ 5 points [7].

### ASA classification

ASA classifications were used to classify patients’ risk burden [20]. Patient classification was performed by the anaesthesiologist, making it an independent evaluation to at least some extent. When using the ASA classification system for risk evaluation, the usual dichotomy is that patients in ASA class 3 or higher have a substantially increased risk for postoperative cardiac and pulmonary complications [28].

### mRS score for assessment of disability/independence

The modified Rankin scale (mRS) was originally used to classify disability/dependence in stroke patients, but it can be used for many other diseases as well, including iNPH [26]. To measure improvement in disability/independence, we constructed the ∆mRS, defined as *pre-operative mRS – post-operative mRS*, where a positive value represents improvement in disability/independence.

### Statistics

Distributions are presented as median and interquartile range (IQR), since the data are not normally distributed (as tested by the Shapiro–Wilk and Anderson–Darling tests). *P* values are two-tailed and are given with three decimals. For the purpose of discussion, we define statistical significance as *p* < 0.01. Statistical methods are indicated in the text. For statistical analyses, JMP (v. 16.2.0, SAS Institute Inc., Cary, North Carolina, USA) was used, through the methods indicated below.

### Ethical approval

The study was approved by the Swedish Ethical Review Authority (No. 2019–02542).

## Results

At the time of extraction, the register contained 3954 patients with an iNPH diagnosis. Of these, 3082 met the inclusion criteria. The median age was 75.2 years (IQR 71.2–79.1). There were 1855 (60.2%) males and 1227 (39.8%) females, with median ages of 75.3 years (IQR 71.2–79.0) and 75.2 years (IQR 71.1–79.4), respectively (*p* = 0.9208 Wilcoxon rank sums). Age was divided into six groups of 5 years, where Group 1 included patients aged 60–64 years, and Group 6 included patients 85 years or older. No statistically significant differences (*p* > 0.01); Dunn with Control for joint ranks (paired)) between sex in the age groups (Appendix 1 Table 1).

**Fig. 1:**
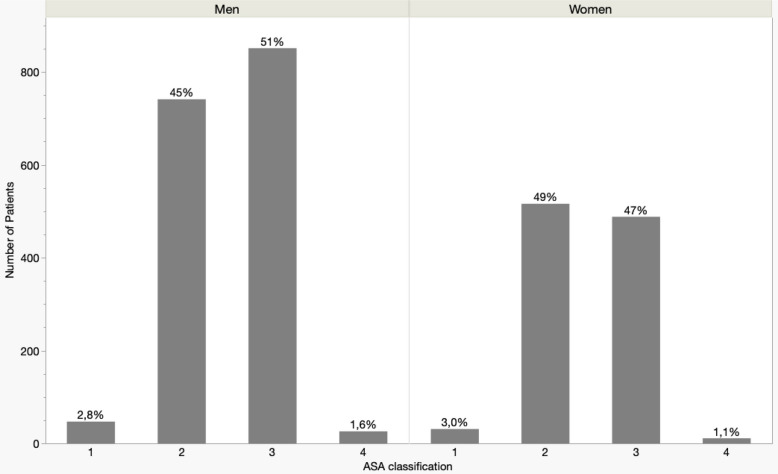
Distribution of sexes by ASA classes 1–4

The median ASA class was 3 (IQR 2–3). There were no statistically significant differences between the sexes (Appendix 2 Fig. 1).

In the total material, the median pre-operative and 3-month post-operative miNPH scores were 56.2 (IQR 42.1–68.8 (*n* = 3082)) and 68.0 (IQR 51.2–79.8 (*n* = 2204)), respectively (*p* < 0.0001, Wilcoxon signed-rank test (*n* = 2204)). At the 12-month follow-up, the median miNPH score was 72.6 (IQR 60.9–83.8 (*n* = 667)); *p* < 0.0001, Wilcoxon signed-rank test (*n* = 667).There were no statistically significant differences (Dunn test with control for joint ranks) in post-operative changes in miNPH score between age groups at the 3- and 12-month follow-ups, regardless of sex (Appendix 3 Table 2).

Overall, 54.0% showed improvement – that is, an increase of ≥ 5 points – in their miNPH score at the 3-month follow-up. At the 12-month follow-up, 60.0% showed improvement (Appendix 4 Table 3). No statistically significant differences between age groups were found (Dunn test with control for joint ranks).

The median pre-operative TUG test result was 24.6 s and that at 3 months was 19.0 s, showing an improvement of 5.6 s (*p* < 0.0001, Wilcoxon signed-rank test); the improvement at 12-month follow-up was 10.7 s (*p* < 0.0001, Wilcoxon signed-rank test), with no difference between sexes (Appendix 5 Table 4).

Analyses of the TUG tests between age groups (Dunn test with controlled ranks) showed no statistical differences.

There were no statistically significant differences in improved TUG outcome for each age group analysed by dichotomized ASA, in any of the calculations made for the TUG test results versus the pre-operative value, nor in the changes at the 3- and 12-month follow-ups. The same calculations were carried out on the miNPH scores and showed no statistically significant differences (Appendix 6 & 7 Tables 5 and 6).

**Fig. 2:**
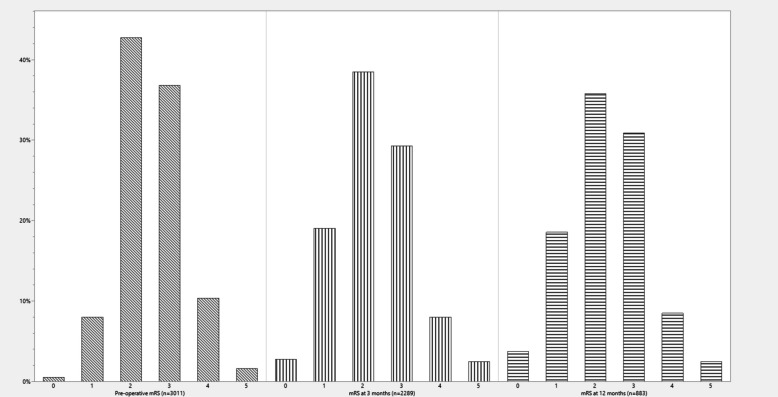
Distribution of mRS

**Fig. 3:**
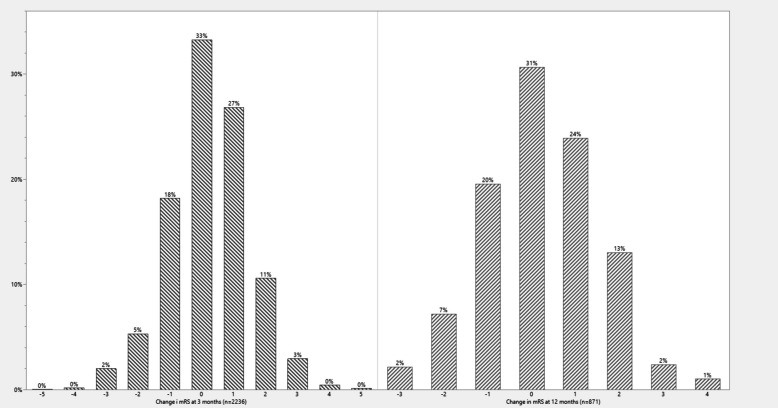
Change in disability (i.e. changes in mRS). Positive values represent improvement

The disability of the patients in the register was described using the mRS scale. The ∆mRS between the pre-operative status and the status at 3 and 12 months was calculated. The mean mRS improved by 0.24 points between the pre-operative status and the follow-up at 3 months (*n* = 2236; *p* < 0.0001, Pearson chi square). The improvement at 12 months was 0.31 (*n* = 965; *p* < 0.0001, Pearson chi square). The difference in mRS at the 3-month follow-up was statistically significant in all the age groups except the two youngest and the oldest (*p* < 0.0001, Wilcoxon signed-rank test). The change in mRS at 12 months was statistically significant in all age groups except the youngest (*p* = 0.0234) (Appendix 8 & 9 Figs. 2 and 3).

Upon dichotomising the outcome so that a change in mRS of 0 or more was regarded as favourable, there was no statistically significant difference in the outcome between the age groups at 3 months (*p* = 0.1477, Pearson chi square). The same was found at 12 months (*p* < 0.3520, Pearson chi square).

## Discussion

The main finding of this population-based study is that age does not influence outcome after shunt surgery in iNPH patients who had three or more domains pre- and post-operatively investigated to form their iNPH score. In fact, this is the only national covering study that has shown that age itself does not affect the outcome of shunt surgery in iNPH patients post-operatively nor at 12 months follow-up. We found no statistically significant differences in outcome, measured as changes in miNPH score at the 3- and 12-month follow-ups, between age groups or in total, nor between sexes. There was also no difference in outcome between age groups when analysing a possible difference in people with a clinically significant improvement – that is, an improvement of 5 or more points on the iNPH scale – whether in the total material or divided by sex. We found no statistically significant difference in either TUG or miNPH when calculating the same follow-up differences, regardless of sex or age group. These results indicate that age does not affect the level of improvement attained by shunt surgery in patients with iNPH.

More specifically, shunt surgery did not have a lesser effect with increasing age, and the outcomes were equally good in the highest age groups. Even though age may be an indicator of risk, our results indicate that decisions on when to perform shunt surgery should not be based on age alone and that older patients may still have a good chance of improvement [2, 3]. This should be taken into consideration when assessing whether a patient is likely to benefit from the suggested (medical or surgical) treatment and when weighing possible benefits against the risk for the patient.

The ASA instrument assesses comorbidities and perioperative risks, which are associated with frailty. The majority of patients included in this study were assessed as ASA class 2 or 3. Interestingly, ASA class was not associated with outcome. Despite the higher perioperative risk of surgery in patients with high ASA class, the outcome was evidently similarly favourable regardless of age. Other measures such as the CFS may better reflect frailty in this patient group. It should be noted that a previous study showed that the waiting time for surgery in patients with iNPH negatively influences outcome [4], with a longer waiting time likely to increase comorbidity among these patients.

As mentioned, the main finding of this population-based study is that age itself does not influence the outcome of shunt surgery for iNPH, despite previous studies suggesting the opposite [2, 19, 24] though not perfectly comparable since not the same parameters were studied. In Bådagård et al. approximately 300 patients were studied and a connection between age and cerebrovascular burden were drawn. In Reddy et al. all types of hydrocephalus were included making it a non iNPH specific study which this study is. Moreover, in Koo et al. connects frailty and shunt surgery but not specifically age and shunt surgery. Kimura et al. found no correlation between mRS and age [10], which our study confirms. In general, we found a greater improvement in mRS at the 12-month follow-up than at the 3-month follow-up. Interestingly, Takeuchi et al. [25] suggest that improvement, especially for patients over 80 years of age, is seen in the first 3 years, prior to deterioration/recurrence of iNPH symptoms. Although we did not follow the patients for as long a time, we did note an improvement in ∆mRS between 3 and 12 months.

### Limitations

The strength of this study is that it is a population-based study with national coverage of patients from all neurosurgical units in Sweden. One of its weaknesses is that the data has gaps, as may be expected when collecting data from a register. Another weakness could be that a greater number of patients were included pre-operatively than at the 3- and 12-month follow-ups. The loss is 28.5% and 78.4% at 3 and 12 months respectively. When comparing the age, sex, iNPH score, ASA class at each follow up point there is no statistically significant differences between the initial data in the group that was followed up as compared to the group lost to follow-up. One could assume that the reason for the loss of follow-up would be that the followed-up patients were for example younger, healthier (lower ASA score) or had less symptoms of their iNPH (higher iNPH score) or the other way around. This seems not be the case. This lack of differences would, in the opinion of the authors, suggest that there is no systematic error in the follow-up. The results could therefore despite the large loss to follow-up, especially at 12 months, be regarded as trustworthy. We purposely chose to include only those patients with three or more domains in the iNPH scale, since these are the best investigated. However, a bias might be introduced by leaving out patients with fewer than three domains, since a possible reason for the data being incomplete for these individuals might be that they were frailer and more unable to go through a complete investigation. However, there was no difference in either age or sex between those with one or two domains in their pre-operative evaluation and those with three or four domains, and as many as 85% of the patients in the registry had scores for three or more domains available.

## Conclusion

This population-based study shows similarly favourable outcomes across ages, suggesting that there should be no upper age limit for shunt surgery in patients with iNPH.

However, further studies must be conducted to identify comorbidities that would prevent surgeons from recommending patient shunt surgery.

## Data Availability

No datasets were generated or analysed during the current study.

## Appendix 1

Table 1.

**Table 1:** Age distribution of 3082 patients with iNPH

***Age***	**Females**		**Males**		**Total**	
	N	Age Median (IQR)	*N*	Age Median (IQR)	*N*	Age Median (IQR)
*60–64*	54	62.9 (61.9–64.1)	89	63.3 (62.2–64.2)	143	63.2 (62.2–64.2)
*65–69*	201	68.1 (66.5–69.1)	258	68.1 (66.8–69.0)	459	68.1 (66.6–69.0)
*70–74*	342	72.9 (71.6–73.9)	540	72.8 (71.3–74.0)	882	72.8 (71.5–74.0)
*75–79*	360	77.4 (76.3–78.6)	606	77.3 (76.2–78.7)	966	77.3 (76.2–78.7)
*80–84*	213	82.2 (80.8–83.5)	306	82.0 (80.8–83.3)	519	82.1 (80.8–83.3)
> *85*	57	87.2 (85.6–88.2)	56	86.8 (86.1–88.3)	113	86.9 (85.7–88.3)
*Total*	1227	75.2 (71.1–79.4)	1855	75.3 (71.2–79.0)	3082	75.2 (71.2–79.1)

No statistically significant differences (*p* > 0.01) between sex in the age groups using Dunn with control for joint ranks (paired)

## Appendix 2

Figure 1.

## Appendix 3

Table 2.

**Table 2:** Median (IQR) miNPH points pre-operatively and median changes at 3- and 12-month follow-ups

Age (years)	miNPH scores								
	Females			Males			Total		
	Pre-op	3 months vs. pre-op	12 months vs pre-op	Pre-op	3 months vs. pre-op	12 months vs. pre-op	Pre-op	3 months vs. pre-op	12 months vs. pre-op
60–64	64.3 (36.8–77.3) *n* = 54	6.2 (– 21.3–19.1) *n* = 38	18.5 (–3.1–53.9) *n* = 13	52.3 (42.0–70.3) *n* = 89	11.0 (–9.1–26.0) *n* = 57	26.5 (–2.8–40.8) *n* = 22	55.5 (41.3–74.8) *n* = 143	10.0 (–12.8–24.0) *n* = 95	25.5 (–1.7–45.2) *n* = 35
65–69	52.4 (39.2–68.8) *n* = 201	13.9 (–2.3–30.1) *n* = 149	16.5 (7.5–34.5) *n* = 55	61.1 (44.6–72.3) *n* = 258	5.9 (–9.9–23.5) *n* = 180	10.5 (–3.7–30.9) *n* = 56	58.4 (42.0–71.3) *n* = 459	8.7 (–6.1–26.6) *n* = 329	14.0 (0.3–32.0) *n* = 111
70–74	55.1 (41.4–68.2) *n* = 342	8.8 (–6.6–22.9) *n* = 244	19.2 (2.3–33.0) *n* = 84	56.2 (42.8–69.7) *n* = 540	8.8 (–7.0–26.8) *n* = 395	18.5 (–1.0–32.5) *n* = 119	55.6 (42.5–68.9) *n* = 882	8.8 (–6.9–24.7) *n* = 639	19.0 (0.0–32.8) *n* = 203
75–79	56.5 (44.0–67.3) *n* = 360	7.7 (–9.8–24.5) *n* = 241	17.9 (–2.6–34.1) *n* = 78	55.9 (42.0–67.5) *n* = 606	9.4 (–7.1–26.0) *n* = 429	13.4 (–3.5–28.0) *n* = 114	56.0 (42.8–67.4) *n* = 966	9.1 (–7.9–25.1) *n* = 670	15.0 (–3.3–30.0) *n* = 192
80–84	54.3 (41.2–67.3) *n* = 213	6.7 (–12.0–28.3) *n* = 158	5.8 (–4.9–20.5) *n* = 42	55.6 (42.0–67.3) *n* = 306	11.9 (–5.3–29.1) *n* = 230	9.8 (–4.7–34.4) *n* = 57	55.3 (41.9–67.3) *n* = 519	10.0 (–7.5–28.7) *n* = 388	9.3 (–4.0–28.5) *n* = 99
> 85	58.2 (46.6–66.7) *n* = 57	4.5 (–9.9–22.7) *n* = 42	16.5 (1.6–32.6) *n* = 13	59.4 (41.5–70.4) *n* = 56	6.0 (–11.3–27.8) *n* = 41	4.5 (–15.6–30.2) *n* = 14	58.3 (42.7–67.6) *n* = 113	5.0 (–10.3–26.6) *n* = 83	10.1 (–6.0–28.5) *n* = 27
Total	55.5 (41.8–68.2) *n* = 1227	9.0 (–7.5–24.6) *n* = 872	16.5 (–0.4–32.9) *n* = 285	56.3 (42.5–68.8) *n* = 1855	8.9 (–7.5–26.5) *n* = 1332	13.9 (–3.1–30.3) *n* = 382	56.2 (42.1–68.8) *n* = 3082	9.0 (–7.5–26.0) *n* = 2204	15.0 (–1.8–32.0) *n* = 667

No statistically significant differences between age groups at 3- and 12-month follow-ups compared with pre-operative (Dunn test with control for joint ranks (pared))

## Appendix 4

Table 3.

**Table 3:** Improvement in miNPH score points in those who improved ≥ 5 points or more in miNPH scale score. Values given as median (IQR)

Age	Females		Males		Total	
	3 months vs pre-op	12 months vs pre-op	3 months vs pre-op	12 months vs pre-op	3 months vs pre-op	12 months vs pre-op
60–64	18.8 (12.2–26.6) *n* = 19	48.6 (22.0–62.7) *n* = 8	24.6 (15.3–33.2) *n* = 32	33.5 (25.5–46.2) *n* = 15	22.6 (14.3–33.2) *n* = 51	34.5 (25.5–50.7) *n* = 23
65–69	22.5 (15.0–34.8) *n* = 95	23.1 (13.0–36.8) *n* = 44	22.6 (10.2–33.3) *n* = 96	23.3 (12.0–34.5) *n* = 35	22.5 (12.8–34.3) *n* = 191	23.3 (13.0–35.3) *n* = 79
70–74	21.0 (12.0–32.5) *n* = 139	24.3 (17.7–37.2) *n* = 60	24.3 (12.5–34.8) *n* = 225	26.3 (17.2–37.5) *n* = 84	22.5 (12.2–33.9) *n* = 364	25.8 (17.3–37.2) *n* = 144
75–79	23.5 (15.0–36.0) *n* = 131	27.0 (17.2–40.9) *n* = 53	21.7 (12.0–37.4) *n* = 257	23.2 (13.4–35.4) *n* = 76	22.2 (12.9–37.0) *n* = 388	25.0 (14.7–37.1) *n* = 129
80–84	26.7 (15.6–40.2) *n* = 85	19.9 (14.0–42.1) *n* = 23	24.5 (15.3–35.5) *n* = 143	28.3 (13.3–45.9) *n* = 34	25.3 (15.4–38.3) *n* = 228	22.3 (13.9–43.9) *n* = 57
> 85	20.5 (10.1–33.6) *n* = 21	24.2 (11.6–38.2) *n* = 10	25.0 (11.8–43.0) *n* = 22	32.9 (13.7–44.4) *n* = 6	23.5 (12.1–33.9) *n* = 43	26.1 (13.2–39.8) *n* = 16
Total	22.5 (14.0–36.0) *n* = 490	24.5 (15.0–37.9) *n* = 198	23.3 (12.5–35.3) *n* = 775	26.2 (14.5–38.3) *n* = 250	23.1 (13.1–35.5) *n* = 1265	25.7 (14.8–38.1) *n* = 448

No statistically significant differences between age groups at 3- and 12-month follow-ups compared with pre-operative (Dunn test with control for joint ranks (paired))

## Appendix 5

Table 4.

**Table 4:** Pre-operative median (IQR) TUG time (s) and reduction in TUG time (s) (i.e. improvement) at 3- and 12-month follow-ups

Age (years)	Females			Males			Total		
	Pre-op	Pre-op vs. 3 months	Pre-op vs. 12 months	Pre-op	Pre-op vs. 3 months	Pre-op vs. 12 months	Pre-op	Pre-op vs. 3 months	Pre-op vs. 12 months
60–64	17.0 (13.0–32.0) *n* = 29	3.9 (–1.2–18.8) *n* = 22	15.3 (1.4–22.2) *n* = 4	17.5 (13.0–30.4) *n* = 54	0.7 (–3.5–15.3 *n* = 31	6.5 (2.1–23.0) *n* = 8	17.0 (13.0–31.0) *n* = 83	3.0 (–1.6–15.9) *n* = 53	10.3 (2.1–22.2) *n* = 12
65–70	20.0 (14.4–29.5) *n* = 145	6.0 (0.0–15.0) *n* = 99	7.1 (1.2–15.3) *n* = 30	17.0 (13.1–25.0) *n* = 172	2.8 (–3.0–8.1) *n* = 110	6.0 (–0.5–13.2) *n* = 22	18.5 (13.6–27.0) *n* = 317	3.5 (–1.5–11.0) *n* = 209	6.1 (0.4–13.9) *n* = 52
71–74	19.0 (15.0–27.1) *n* = 262	3.9 (–2.0–11.5) *n* = 171	6.0 (–3.3–24.5) *n* = 33	19.0 (14.4–27.1) *n* = 385	3.3 (–2.1–11.0) *n* = 252	7.1 (–2.5–19.0) *n* = 55	19.0 (14.6–27.0) *n* = 647	3.5 (–2.0–11.3) *n* = 423	6.5 (–2.9–19.8) *n* = 88
75–79	19.0 (14.7–27.0) *n* = 251	3.3 (–2.5–11.0) *n* = 158	4.0 (–1.5–19.8) *n* = 33	18.2 (14.0–27.0) *n* = 443	3.0 (–3.0–12.0) *n* = 295	1.0 (–4.5–16.7) *n* = 53	18.6 (14.0–27.0) *n* = 694	3.0 (–3.0–11.4) *n* = 452	3.2 (–2.9–19.1) *n* = 86
80–84	19.9 (14.1–27.8) *n* = 169	2.0 (–2.8–10.8) *n* = 106	2.0 (–5.3–10.5) *n* = 22	20.0 (15.0–30.0) *n* = 219	5.0 (–3.6–13.0) *n* = 142	2.0 (–4.0–12.0) *n* = 27	20.0 (14.6–29.0) *n* = 388	3.3 (–3.0–12.0) *n* = 248	2.0 (–4.0–11.5) *n* = 47
> 85	18.4 (13.0–22.8) *n* = 46	0.0 (6.4–7.8) *n* = 33	17.5 (7.6–39.8) *n* = 4	21.6 (14.7–27.5) *n* = 45	6.6 (–4.7–12.0) *n* = 32	9.2 (–1.8–15.3) *n* = 8	19.0 (14.0–25.5) *n* = 91	3.0 (–5.4–9.0) *n* = 65	11.6 (6.1–18.0) *n* = 12
Total	19.0 (14.2–27.1) *n* = 902	3.6 (–2.3–11.0) *n* = 589	5.8 (–1.0–18.9) *n* = 124	19.0 (14.0–27.4) *n* = 1318	3.0 (–3.0–11.2) *n* = 861	5.0 (–2.3–14.0) *n* = 173	19.0 (14.0–27.3) *n* = 2220	3.3 (–2.9–11.0) *n* = 1450	5.0 (–2.0–15.9) *n* = 297

No statistically significant p values when using Dunn test with control for joint ranks (paired)

## Appendix 6

Table 5.

**Table 5:** Median (IQR) reduction (i.e. improvement) in TUG (s) in respective age group and in the total study group by dichotomised ASA classification

Age	ASA-class	Total		
		Pre-op	Pre-op vs. 3 months	Pre-op vs. 12 months
60–64	1–2	18.8 (14–31.0) *n* = 31	4.6 (–0.8–17.6) *n* = 20	11.5 (1.7–23.3) *n* = 7
	3–4	15.6 (11.8–27.2) *n* = 42	1.0 (–2.6–13.0) *n* = 25	3.6 (0.1–10.4) *n* = 4
65–69	1–2	19.0 (14.1–27.8) *n* = 136	5.0 (–1.5–14.0) *n* = 85	11.0 (–0.8–17.1) *n* = 22
	3–4	16.5 (13.0–24.5) *n* = 137	3.0 (–1.1–9.1) *n* = 92	4.6 (–0.6–13.6) *n* = 24
70–74	1–2	20.7 (15.0–30.0) *n* = 251	3.2 (–3.1–13.0) *n* = 165	7.5 (0.5–22.0) *n* = 43
	3–4	18.2 (14.0–25.0) *n* = 308	3.5 (–1.5–9.1) *n* = 195	5.0 (–3.8–11.5) *n* = 38
75–79	1–2	19.0 (14.5–29.0) *n* = 294	4.0 (–3.0–13.0) *n* = 186	4.5 (–1.0–21.7) *n* = 32
	3–4	18.2 (13.6–26.0) *n* = 305	2.0 (–3.0–10.0) *n* = 204	1.0 (–5.0–16.7) *n* = 41
80–84	1–2	20.2 (14.9–28.5) *n* = 138	6.0 (–2.5–14.4) *n* = 91	2.0 (–13.4–13.4) *n* = 14
	3–4	21.0 (14.5–29.0) *n* = 203	2.0 (–3.9–10.8) *n* = 123	4.5 (–1.0–12.0) *n* = 27
> 85	1–2	19.5 (13.4–27.4) *n* = 28	–2.1 (–9.0–6.7) *n* = 20	13.5 (8.3–18.7) *n* = 2
	3–4	19.6 (13.5–26.0) *n* = 51	4.0 (–4.4–11.4) *n* = 36	13.0 (0.3–18.5) *n* = 9
Total	1–2	20.0 (14.6–29.0) *n* = 878	4.0 (–3.0–12.9) *n* = 567	7.9 (–0.7–19.0) *n* = 120
	3–4	18.7 (14.0–26.0) *n* = 1046	3.0 (–2.2–10.0) *n* = 675	4.5 (–3.0–13.0) *n* = 143

## Appendix 7

Table 6.

**Table 6:** Median (IQR) improvement in miNPH score by age groups and dichotomised ASA for those improved ≥ 5 points in miNPH score

Age	ASA class	Total		
		Pre-op	Pre-op vs. 3 months	Pre-op vs. 12 months
60–64	1–2	52.1 (40.5–70.1) *n* = 64	22.9 (18.1–28.8) *n* = 24	36.5 (20.3–49.1) *n* = 12
	3–4	58.2 (41.2–77.4) *n* = 62	22.4 (11.4–36.3) *n* = 22	32.9 (25.4–52.5) *n* = 10
65–69	1–2	58.9 (42.3–70.8) *n* = 216	25.3 (12.5–38.3) *n* = 91	21.0 (13.2–35.8) *n* = 32
	3–4	60.3 (42.5–72.4) *n* = 184	20.4 (12.3–30.8) *n* = 77	22.4 (12.4–34.3) *n* = 34
70–74	1–2	56.0 (42.0–69.8) *n* = 383	23.4 (13.8–33.9) *n* = 152	26.3 (18.5–40.0) *n* = 67
	3–4	56.0 (43.6–69.3) *n* = 388	21.0 (11.5–31.5) *n* = 163	25.5 (15.8–37.3) *n* = 63
75–79	1–2	57.4 (43.7–69.2) *n* = 428	22.2 (13.6–36.2) *n* = 168	25.3 (17.9–41.8) *n* = 46
	3–4	56.1 (42.8–67.7) *n* = 420	21.3 (11.7–37.1) *n* = 166	22.6 (13.3–32.8) *n* = 62
80–84	1–2	56.3 (42.0–72.3) *n* = 207	26.8 (16.0–39.4) *n* = 89	22.5 (13.5–45.0) *n* = 21
	3–4	55.3 (42.0–66.5) *n* = 259	21.7 (13.6–37.2) *n* = 112	21.6 (12.0–44.8) *n* = 31
> 85	1–2	57.2 (41.7–69.2) *n* = 37	33.3 (25.3–47.5) *n* = 12	42.6 (28.5–56.8) *n* = 2
	3–4	58.4 (41.3–67.3) *n* = 63	17.7 (7.5–31.0) *n* = 27	24.2 (11.6–38.7) *n* = 14
Total	1–2	56.9 (42.4–69.9) *n* = 1335	23.5 (14.7–35.9) *n* = 536	25.7 (16.6–41.3) *n* = 180
	3–4	56.3 (42.5–68.8) *n* = 1376	21.0 (11.8–33.5) *n* = 567	24.2 (13.9–36.0) *n* = 214

## Appendix 8

Figure 2.

## Appendix 9

Figure 3.
